# Computational plant bioscience at BGRS\SB-2016: introductory note

**DOI:** 10.1186/s12870-016-0923-0

**Published:** 2016-11-16

**Authors:** Yuriy L. Orlov, Ancha V. Baranova, Elena A. Salina

**Affiliations:** 1 Institute of Cytology and Genetics SB RAS, Lavrentyeva, 10, 630090 Novosibirsk, Russia; 2 Novosibirsk State University, 630090 Novosibirsk, Russia; 3 School of Systems Biology, George Mason University, Fairfax, VA 22030 USA

In this Special Issue, we feature materials presented at the Bioinformatics of Genome Regulation and Structure\Systems Biology (BGRS\SB) multi-conference (http://conf.bionet.nsc.ru/bgrssb2016) and earlier workshop on Plant Genetics and Genomics (http://conf.bionet.nsc.ru/plant-food/).

BGRS\SB’2016 took place August 29 - September 2, 2016 in Novosibirsk, Russia, together with the Young Scientist School in Systems Biology and Bioinformatics (SBB) (http://conf.bionet.nsc.ru/sbb2016/en/). The BGRS conference series on bioinformatics was initiated by Academician N.A. Kolchanov, andfter the first relatively small international meeting in 1998, BGRS/SB was held biannually by the Institute of Cytology and Genetics of Siberian Branch of the Russian Academy of Sciences (ICG SB RAS) in Novosibirsk Akademgorodok. 2016 marks the tenth anniversary of the BGRS series; this year the conference gathered more than 500 participants from different countries and included several symposia covering topics in systems biology. Full coverage of the BGRS\SB-2016 Proceedings, with the abstracts for all lectures, oral presentations and posters, including these presented at “Bioinformatics and Systems Biology of Plants” section, are available at http://www.bionet.nsc.ru/files/2016/conference/BGRS2016.pdf.

Over the last two decades, the BGRS/SB series gained a reputation as a prime meeting venue for biologists, computer scientists, physicists, mathematicians and biochemists working in the interdisciplinary systems biology field, now focusing more on computational plant biology. The section was dedicated to genomic and post-genomic approaches to analysis of the structural and functional organization of the genome, and the integration of acquired knowledge into plant systems biology.

A key goal of plant genetics research is the search for fundamental knowledge useful for breeding new, highly productive cultivars with characteristics that have implications for end-product quality. In this important avenue of study, this Special Issue starts with two articles investigating the genetics of wheat, one of the most important food stocks in the world [1, 2]. Both works center on the function of *VRN1* (Vernalization) gene. The paper by Irina Konopatskaia and colleagues “*VRN1* genes variability in tetraploid wheat species with a spring growth habit” considers association of the growth habits of 228 tetraploid wheat species and allelic variants of *VRN1* genes [1]. In “The occurrence of spring forms in tetraploid Timopheevi wheat is associated with variation in the first intron of the *VRN-A1* gene,” Shcherban and co-authors discuss the promoter region of the *VRN-A1* gene in *T. araraticum* and *T. timopheevii* tetraploid species [2]. Another article in this issue features analysis of hexaploid bread wheat (*Triticum aestivum L*., AABBDD) [3] – another wheat species with significant agricultural value.

Shmakov et al. “Identification of nuclear genes controlling chlorophyll synthesis in barley by RNA-seq” [4] used transcript sequencing to identify genes responsible for chlorophyll synthesis in barley, another important crop plant.

Work by Dmitriev et al. “Gene expression profiling of flax (*Linum usitatissimum* L.) under edaphic stress” [5] presents gene expression in flax. Cultivated flax (*Linum usitatissimum* L.) is widely used for production of textile, food, chemical and pharmaceutical goods. High-throughput sequencing of the transcriptome of flax plants in stress conditions allowed identification of genes differentially expressed in phosphate-deficient and excess nutrition conditions.

The paper by Sugawara et al. “Expression of an extracellular ribonuclease gene increases resistance to Cucumber mosaic virus in tobacco” [6] considers a tobacco model example of plant defense against pathogens. The authors demonstrated that elevated levels of extracellular RNAse activity resulted in increased resistance to *Cucumber mosaic virus* in tobacco plants.

Work by Ravin and colleagues, “The loss of photosynthetic pathways in the plastid and nuclear genomes of the non-photosynthetic mycoheterotrophic eudicot *Monotropa hypopitys,*” [7] presents research on the plastid genome of obligate mycoheterotrophic plant belonging to the family *Ericaceae*. Using genome sequencing the authors found that the *M. hypopitys* plastid genome is among the most functionally reduced ones characteristic of obligate non-photosynthetic parasitic species.

Finally we have to acknowledge a number of organizations that supported BGRS conference series and plant biology studies presented here: Institute of Cytology and Genetics SB RAS (www.bionet.nsc.ru/), the Novosibirsk State University (www.nsu.ru/), the Federal Agency for Scientific Organizations (fano.gov.ru), the Siberian Branch of the Russian Academy of Sciences (http://www.sbras.ru/en/) and the Vavilov Society of Geneticists and Breeders (http://www.bionet.nsc.ru/vogis/; http://vogis.org/).

This series of plant biology meetings will continue in coming years – we are glad to announce PlantGen-2017 conference in Kazakhstan (http://primerdigital.com/PlantGen2017/en/) and BGRS\SB-2018 conference in Novosibirsk. We welcome all the readers to participate in these meetings.

Overall this thematic issue on plant biology research continues traditions of systems biology studies in plant in previous BMC Series supplement issues http://bmcplantbiol.biomedcentral.com/articles/supplements/volume-16-supplement-1. We would also like to mention our ongoing collaboration with BioMed Central on full-text thematic issues reflecting research milestones covered by of this conference series. In the past BioMed Central had published several special issues highlighting materials presented at the BGRS conference, including issues of BMC Genomics (http://www.biomedcentral.com/bmcgenomics/supplements/15/S12), BMC Evolutionary Biology (http://www.biomedcentral.com/bmcevolbiol/supplements/15/S1), BMC Genetics (http://www.biomedcentral.com/bmcgenet/supplements/16/S1) and BMC Systems Biology (http://www.biomedcentral.com/bmcsystbiol/supplements/9/S2), see also SBB issue [8]. Other papers from BGRS\SB-2016 are included in Supplementary Issues to the journals of BMC Series, namely BMC Plant Biology, BMC Genomics, BMC Genetics, BMC Systems Biology and BMC Bioinformatics.

In addition, special issues on bioinformatics were published at Journal of Bioinformatics and Computational Biology (http://www.worldscientific.com/toc/jbcb/13/01) [9] and “Vavilov journal of selection and breeding” (http://www.bionet.nsc.ru/vogis/2014-year/18-4-2/) (in Russian). The Plant biology meetings in Novosibirsk are part of PlantGen series of meetings, also presented in a BioMed Central thematic issue http://bmcplantbiol.biomedcentral.com/articles/supplements/volume-16-supplement-1. Materials of the BGRS conference events and plant genetics workshops are published in a special issue of “Vavilov journal of selection and breeding” http://www.bionet.nsc.ru/vogis/2014-year/18-4-1/. The Proceedings of the 3rd International Conference “Plant Genetics, Genomics, Bioinformatics and Biotechnology” are also available (http://csl.isc.irk.ru/BD/Books/Plant%20genetics.pdf).
